# Gingival cyst of the adult

**DOI:** 10.4322/acr.2023.454

**Published:** 2023-11-13

**Authors:** Maria Emília Mota, Dandara Menezes de Araujo Oliveira, Yuri de Lima Medeiros, Maria Stella Moreira, Rodrigo Nascimento Lopes, Fábio Abreu Alves, Brendo Vinícius Rodrigues Louredo, Pablo Agustin Vargas, José Divaldo Prado

**Affiliations:** 1 Universidade de São Paulo, Faculdade de Odontologia, Departamento de Estomatologia, São Paulo, SP, Brasil; 2 A.C. Camargo Cancer Center, Departamento de Estomatologia, São Paulo, SP, Brasil; 3 Universidade Estadual de Campinas, Faculdade de Odontologia de Piracicaba, Departamento de Diagnóstico Oral, Piracicaba, Brasil

**Keywords:** Cysts, Odontogenic Cysts, Gingival Diseases

## Abstract

The gingival cyst of the adult (GCA) is a rare odontogenic cyst, consisting of 0.3% of all odontogenic cysts. This case report, based on CARE guidelines for case reports, aims to present a case of a 52-year-old female patient with a symptomatic translucent nodule in the upper left anterior gingiva, measuring approximately 6mm. Excisional biopsy was performed, and the histological examination revealed multiple cystic cavities lined by the squamous epithelium of varying thickness with focal areas of nodular thickenings. The presence of clusters of cells with clear cytoplasm within epithelial thickenings was observed. PAS staining was negative in clear cells. The diagnosis of the GCA was established. Despite its rarity, GCA should be considered in the differential diagnosis of gingival lesions. Conservative surgical treatment proved to be effective, with no signs of recurrence.

## INTRODUCTION

The gingival cyst of the adult (GCA) is a rare odontogenic cyst with 157 cases reported until 2019.^1^ The etiology of GCA is uncertain, however, some hypotheses have been suggested such as the derivation from heterotopic glandular elements and epithelial remnants enamel, dental lamina or periodontal ligament. In addition, the possibility of migration of epithelial remnants from the surface epithelium after trauma is discussed.^2-4^

This lesion is characterized by a capsule composed of fibrous connective tissue with mild inflammation coated by non-keratinized epithelium.^5^ Usually, present as nodules in the vestibular gingiva of the mandibular canine and pre-molars of women around the 5th and 6th decades of life.^6^

Hereby, we report a case of a 52-year-old female patient with a GCA in the in the upper left gingiva.

## CASE REPORT

**Figure 1:**
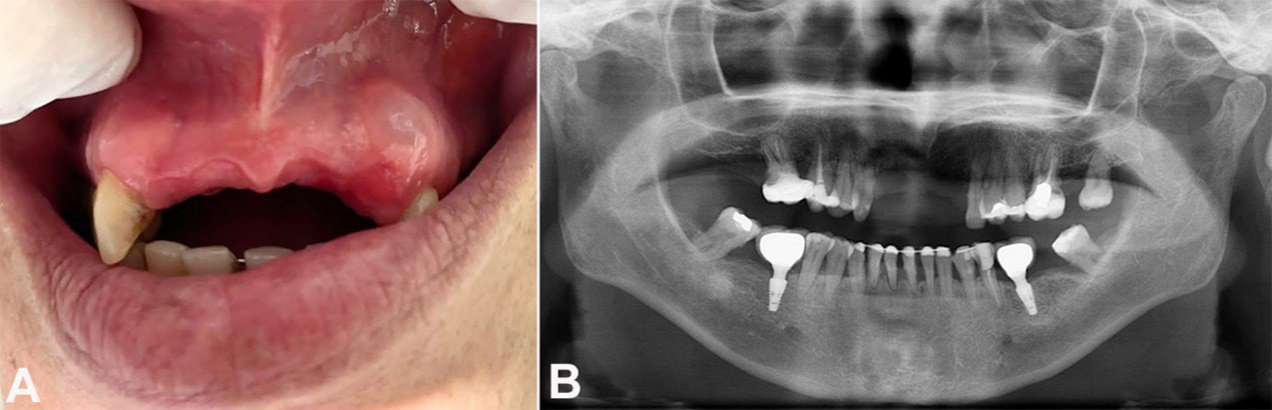
**A -** Gross view of the oral examination showing a translucent nodule in the upper left gingiva, near tooth #11, measuring approximately 6mm**; B -** Panoramic radiographic without evident lesions.

This case report was conducted based on the CARE guidelines for case reports.^7^ A 52-year-old female patient presented to the dentist complaining of a gingival lesion over the last 3 months. The patient denied a history of local trauma. The oral examination revealed an asymptomatic translucent nodule in the upper left gingiva, near tooth #11, measuring approximately 6mm, absent anterior teeth, and generalized gingival resorption (Figure 1A). There were no imaging changes (Figure 1B).

**Figure 2:**
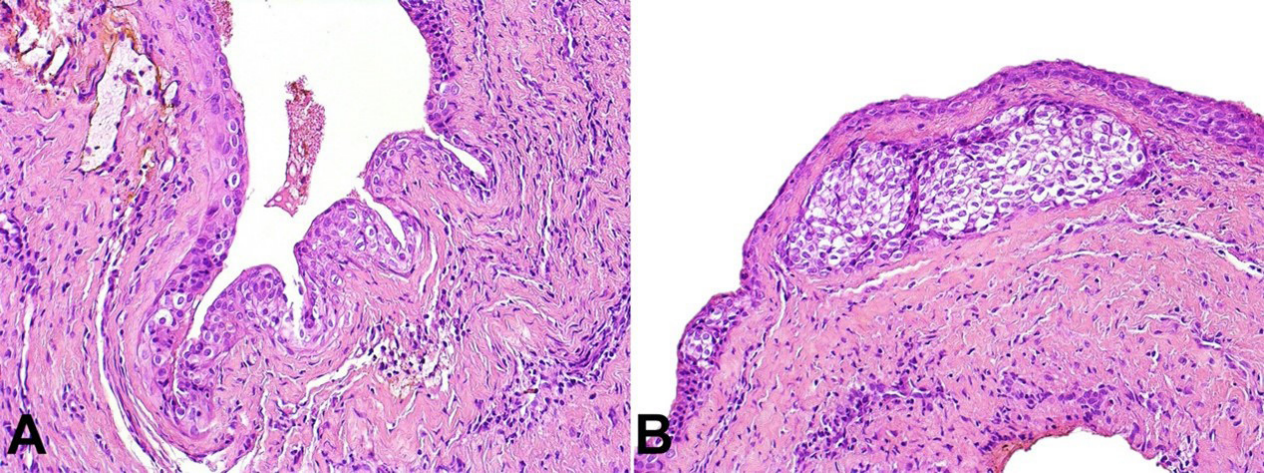
Histopathological findings of the biopsy. **A** and **B -** Multiple cystic cavities lined by squamous epithelium of varying thickness with focal areas of nodular thickenings. The presence of clusters of cells with clear cytoplasm within epithelial thickenings was observed (H&E, 200x).

A surgical excision was performed, and histopathological findings revealed multiple cystic cavities lined by squamous epithelium of varying thickness with focal areas of nodular thickenings. The presence of clusters of cells with clear cytoplasm within epithelial thickenings was observed (Figure 2). PAS staining was negative in the clear cells. The diagnosis of the GCA was established. No signs or symptoms of lesion recurrence were observed at 1-year follow-up.

## DISCUSSION

GCA is a rare odontogenic lesion, constituting 0.3% of all odontogenic cysts, occurring mainly in the mandible.^8,9^ Most lesions occur in the region of lower incisors, canines, and premolars,^2^ and usually affect adults between the fifth and sixth decades of life, with a higher prevalence in women.^6^

GCA usually presents as a firm, well-defined, nodular elevation with a transparent and slightly bluish color. Generally, less than 1 cm in size in the attached gingiva or interdental papilla.^5-6,10^ In our case, the patient presented a classic GCA in the upper left gingiva. Based on the clinical features, the differential diagnoses include: reactive lesions (peripheral giant cell lesions, peripheral ossifying fibroma, pyogenic granuloma), other odontogenic cysts as a lateral periodontal cysts or inflammatory cysts as the radicular cyst.^1,6^ Furthermore, gingival metastases commonly resemble benign nodules.^11^ The GCA is only diagnosed in 50%^12^ to 55%^6^ of the cases.

Among the odontogenic cysts, GCA only originates exclusively from soft tissues.^8^ This cyst has indolent radiographic features,^1^ however, it may cause radiolucent changes due to the pressure atrophy of the underlying alveolar bone.^2^ In some cases, the atrophy can be so intense that it generates a more well-defined lesion similar to those of lateral periodontal cysts.^13^ In the 157 cases found in the literature, no reports of bone expansion, cortical bone perforation, tooth displacement or resorption caused by GCA were observed. In 38 cases (24.2%), bone erosion was observed.^1^ A series of 20 cases^12^ demonstrated the presence of radiographic alterations in only two patients. Our case did not present any bone involvement.

Histologic features of GCA include a non-inflammatory fibrous capsule lined by a non-keratinized thin epithelium, eventually with areas of thickening.^6,10^ It may also show remnants of glycogen-rich clear cells from the dental lamina, cystic enlargement, and degeneration with the formation of microcysts.^2^

Surgical excision was the treatment in 96.5% of cases reported in the literature. Other forms of therapy included curettage (2.7%) and enucleation (0.9%).^1^ Follow-up ranged from 1 to 92 months. Only 1 case of GCA recurrence after 7 years of the lesion’s excision was found. However, it is not possible to confirm whether it was a true recurrent cyst or a second lesion in the same location of the excision.^13^ Generally, recurrence is not expected after surgical excision of the lesion with minimal margins.

## CONCLUSION

Despite its rarity, the GCA should be considered in the differential diagnosis of gingival lesions. A correct diagnosis can exclude lesions with a worse prognosis or that require more aggressive therapy. In the presented case, conservative surgical excision proved to be effective, with no signs of recurrence.
